# An Exact Approach to Elimination of Leakage in a Qubit Embedded in a Three-level System

**DOI:** 10.1038/s41598-019-47479-9

**Published:** 2019-07-30

**Authors:** Yifan Sun, Jun-Yi Zhang, Lian-Ao Wu

**Affiliations:** 1 State Key Laboratory of Magnetic Resonance and Atomic and Molecular Physics, Wuhan Institute of Physics and Mathematics, Chinese Academy of Sciences, Wuhan, 430071 China; 2 Department of Theoretical Physics and History of Science, The Basque Country University (EHU/UPV), PO Box 644, 48080 Bilbao, Spain; 3 Ikerbasque, Basque Foundation for Science, 48011 Bilbao, Spain; 4 Beijing Key Laboratory of Nanophotonics & Ultrafine Optoelectronic Systems, School of Physics, Beijing Institute of Technology, 100081 Beijing, China

**Keywords:** Quantum information, Qubits

## Abstract

Leakage errors damage a qubit by coupling it to other levels. Over the years, several theoretical approaches to dealing with such errors have been developed based on perturbation arguments. Here we propose a different strategy: we use a sequence of finite rotation gates to exactly eliminate leakage errors. The strategy is illustrated by the recently proposed charge quadrupole qubit in a triple quantum dot, where there are two logical states to encode the qubit and one leakage state. We found an *su*(2) subalgebra in the three-level system, and by using the subalgebra we show that ideal Pauli *x* and *z* rotations, which are universal for single-qubit gates, can be generated by two or three propagators. In addition, the magnitude of detuning fluctuation can be estimated based on the exact solution.

## Introduction

The physical realization of quantum computer poses an unprecedented challenge to our capabilities of controlling the dynamics of quantum systems. While there have been many attempts to overcome this challenge, the perfect controllability of semiconducting quantum dots makes them promising candidates for universal quantum computation^1–6^. A universal quantum computer is the ultimate information processor in modern quantum technology, which uses quantum bits (qubits) and quantum circuits to perform computations. A qubit consists of an idealized pair of orthonormal quantum states. However, this idealization neglects other states which are typically present and can mix with those encoding the qubit. Such mixing is termed as *leakage*. Leakage may be the result of the application of gate operations, or induced by system-bath interactions^7–13^. Several strategies for combating the leakage errors have been developed for different systems, in particular the semiconducting qubit setup which is the main subject of this work, including analytic pulse shaping^10,14^ and optimal quantum control^15,16^. A general leakage-elimination method has also been presented for removing such errors by using simple decoupling and recoupling pulse sequences of the leakage elimination operator (LEO)^17^. Nonperturbative LEO was recently introduced for nonideal composite pulses, with emphasis on application of three-level nitrogen-vacancy centers^18^. It is shown that, for a three-level system, the effectiveness of LEOs does not depend on the details of the composite pulses but on the integral of the pulse sequence in the time domain. Recent studies show a significant advantage of a three-level system embedded in a triple quantum dot, which is associated with a decoherence-free subspace (DFS) of a charge quadrupole qubit^19^. The leakage errors are caused by noise and could be reduced by smoothly-varying short control pulses which are experimentally feasible. The system is modelled by two logical states and a leakage state coupled to one of them. Using perturbation technique and the quasistatic noise approximation, the leakage errors of single qubit operations can be suppressed by simple pulse sequences up to the sixth order in noise amplitude. While it is simple and efficient, the approach needs additional well-controlled pulses, and is only valid for small-amplitude noise. These requirements may not be well satisfied during gate operations, especially when the strength and time-dependence of noise are not negligible in comparison with other control parameters.

Here we consider exactly the same setting as in ref.^19^, but we are able to solve the inversion exactly by going beyond the first order approximation of that references, by using a simple sequence or circuit of finite rotations (gates). The coupling strength between the logical state and the leakage state is assumed to be static during the operation time, which is experimentally feasible for semiconducting quantum dots^20–22^. In comparison with the previous work based on the setup^19^, our solution is not restricted by the magnitude of the fluctuation and provides a clear view of the relation between the fluctuation and the error. In a more practical manner, we apply the approximation in the case when fluctuation is small and evaluate the corresponding error scales, supported by our numerical analysis. Moreover, our exact solution itself also provides an option to reckon noise strength.

## Brief Introduction of the Model

**Figure 1:**
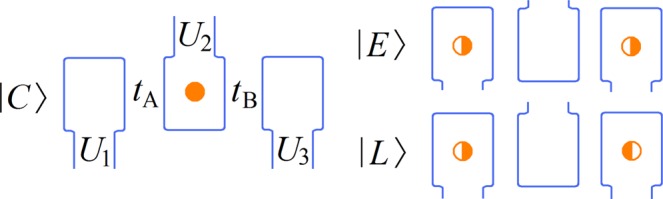
Schematic diagram of CQ qubits. Adjacent quantum dots are represented in blue. *U*_1,2,3_ are on-site potentials, and *t*_A_(*t*_B_) is the coupling between the left(right) and middle dots. Logical states are denoted by |*C*〉, |*E*〉 and leakage state by |*L*〉. The orange dots are electrons, where the full filled one means a single electron and the half filled ones represent the electron being in an equal superposition of |0〉 and |1〉.

We start with a model Hamiltonian represented in the basis spanned by two logical states and one leakage state^19^,

1 $$H={H}_{{\rm{z}}}+{H}_{{\rm{x}}}+{H}_{{\rm{leak}}},$$

with

$$\begin{array}{c}{H}_{{\rm{z}}}=\frac{{\varepsilon }_{{\rm{q}}}}{2}(\begin{array}{ccc}1 & 0 & 0\\ 0 & -1 & 0\\ 0 & 0 & -\zeta \end{array}),{H}_{{\rm{x}}}=g(\begin{array}{ccc}0 & 1 & 0\\ 1 & 0 & 0\\ 0 & 0 & 0\end{array}),\,{\rm{a}}{\rm{n}}{\rm{d}}\,\,{H}_{{\rm{l}}{\rm{e}}{\rm{a}}{\rm{k}}}=\xi (\begin{array}{ccc}0 & 0 & 0\\ 0 & 0 & 1\\ 0 & 1 & 0\end{array}),\end{array}$$

where we use the same notations as in the ref.^19^. *ε*_q_ and *g* are independent control parameters for rotations with respect to the *z* and *x* directions. *H*_leak_ stands for a coupling between the leakage state and one of the logical states, and *ζ* is the scaled leakage state energy in the absence of coupling^23,24^. A charge quadrupole (CQ) qubit is formed in three adjacent semiconducting quantum dots sharing a single electron and is embedded in the localized charge basis {|100〉, |010〉, |001〉}, where the basis states denote the electron being in the first, second or the third dot, respectively. The system Hamiltonian reads

2 $${H}_{{\rm{C}}{\rm{Q}}}=(\begin{array}{ccc}{\varepsilon }_{{\rm{d}}} & {t}_{{\rm{A}}} & 0\\ {t}_{{\rm{A}}} & {\varepsilon }_{{\rm{q}}} & {t}_{{\rm{B}}}\\ 0 & {t}_{{\rm{B}}} & -{\varepsilon }_{{\rm{d}}}\end{array})+\frac{{U}_{1}+{U}_{3}}{2},$$

where *U*_1,2,3_ are the on-site potentials for the three dots. *t*_A,B_ are tunnel couplings between adjacent dots, and *ε*_d_ = (*U*_1_ − *U*_3_)/2 (*ε*_q_ = *U*_2_ − (*U*_1_ + *U*_3_)/2) denotes the dipolar (quadrupolar) detuning parameter. A new set of bases consisting of logical qubtis |*C*〉, |*E*〉 and a leakage state |*L*〉 is defined by^19,20^

3 $$|C\rangle =|010\rangle ,\,|E\rangle =\frac{|100\rangle +|001\rangle }{\sqrt{2}},\,|L\rangle =\frac{|100\rangle -|001\rangle }{\sqrt{2}},$$

and a schematic diagram is presented in Fig. 1. The Hamiltonian in the new basis is transformed into

4 $${\mathop{H}\limits^{ \sim }}_{{\rm{C}}{\rm{Q}}}=(\begin{array}{ccc}\frac{{\varepsilon }_{{\rm{q}}}}{2} & \frac{{t}_{{\rm{A}}}+{t}_{{\rm{B}}}}{\sqrt{2}} & \frac{{t}_{{\rm{A}}}-{t}_{{\rm{B}}}}{\sqrt{2}}\\ \frac{{t}_{{\rm{A}}}+{t}_{{\rm{B}}}}{\sqrt{2}} & -\frac{{\varepsilon }_{{\rm{q}}}}{2} & {\varepsilon }_{{\rm{d}}}\\ \frac{{t}_{{\rm{A}}}-{t}_{{\rm{B}}}}{\sqrt{2}} & {\varepsilon }_{{\rm{d}}} & -\frac{{\varepsilon }_{{\rm{q}}}}{2}\end{array}),$$

where a term proportional to the identity has been dropped. ${\tilde{H}}_{{\rm{CQ}}}$ is reduced to Eq. (1) under the conditions of *ζ* = 1, *ξ* = *ε*_d_, and $g=({t}_{{\rm{A}}}+{t}_{{\rm{B}}})/\sqrt{2}$. In case that *t*_A_ = *t*_B_ and *ε*_d_ = 0 are satisfied, ${\tilde{H}}_{{\rm{CQ}}}$ supports a decoherence-free subspace against uniform electric field fluctuations^20^.

In the triple quantum dot system, *ε*_d_ corresponds to an average dipolar detuning control parameter. Although *ε*_d_ is set to be zero, its fluctuation *δε*_d_ breaks the DFS and causes leakage. It has been shown that the fluctuation of quadrupolar detuning control parameter is smaller than *δε*_d_ and is thus neglected. Now we focus on the influence of *δε*_d_ on the CQ qubit operations. Noise spectrum of *δε*_d_ is dominated by low-frequency fluctuations which are slow in comparison with gate operations^21,22^. Therefore *δε*_d_ is assumed to remain constant during a given gate operation^19,23^. As a result, unitary operators for *x* and *z* rotations can be given by

5 $$\begin{array}{c}{U}_{{\rm{x}}}(g,\delta {\varepsilon }_{{\rm{d}}},\theta )=\exp \{-i[{H}_{{\rm{x}}}(g)+{H}_{{\rm{leak}}}(\delta {\varepsilon }_{{\rm{d}}})]\theta /2g\},\\ {U}_{{\rm{z}}}({\varepsilon }_{{\rm{q}}},\delta {\varepsilon }_{{\rm{d}}},\phi )=\exp \{-i[{H}_{{\rm{z}}}({\varepsilon }_{{\rm{q}}})+{H}_{{\rm{leak}}}(\delta {\varepsilon }_{{\rm{d}}})]\phi /{\varepsilon }_{{\rm{q}}}\},\end{array}$$

with arbitrary angles *θ* and *φ*. In the bang-bang limit where the control pulses switch instantaneously between two values, the angles are associated with the corresponding bang-bang gate time intervals *t*_*z*_ and *t*_*x*_, which are *θ* = *t*_*x*_(2*g*/*ℏ*), *φ* = *t*_*z*_(*ε*_q_/*ℏ*). As shown by Eq. (5), rotation operators are obviously *polluted* by *δε*_d_. Below, we will explain our exact solution to this problem.

## Finite Rotations and Exact Elimination of Leakage

To suppress the fluctuation *δε*_d_ in *U*_x_(*g*, *δε*_d_, *θ*), we start with the following three matrices

$$\begin{array}{c}{M}_{1}=(\begin{array}{ccc}0 & 1 & 0\\ 1 & 0 & 0\\ 0 & 0 & 0\end{array}),{M}_{2}=(\begin{array}{ccc}0 & 0 & 0\\ 0 & 0 & 1\\ 0 & 1 & 0\end{array}),\,{\rm{a}}{\rm{n}}{\rm{d}}\,\,{M}_{3}=(\begin{array}{ccc}0 & 0 & -i\\ 0 & 0 & 0\\ i & 0 & 0\end{array}).\end{array}$$

It can be shown that their commutation relations satisfy

$$\begin{array}{l}[{M}_{1},{M}_{2}]=i{M}_{3},[{M}_{2},{M}_{3}]=i{M}_{1},[{M}_{3},{M}_{1}]=i{M}_{2},\end{array}$$

indicating that these operators generate an *su*(2) algebra. An arbitrarily given finite rotation can be represented in an exponential form^25^

6 $$\exp [i({\gamma }_{1}{M}_{1}+{\gamma }_{2}{M}_{2}+{\gamma }_{3}{M}_{3})],$$

where *γ*_1_, *γ*_2_, *γ*_3_ are three continuous parameters and a linear combination of *M*_*i*_(*i* = 1, 2, 3) indicates a specific rotation axis and the corresponding angle. On the other hand, the finite rotation can also be expressed by three Euler’s angles *ϕ*_1_, *ϕ*_2_ and *ϕ*_3_,

7 $$\exp (i{\varphi }_{1}{M}_{2})\exp (i{\varphi }_{2}{M}_{1})\exp (i{\varphi }_{3}{M}_{2}).$$

The relation between the two sets of parametrizations can be found by setting

8 $$\begin{array}{l}\exp (i{\varphi }_{1}{M}_{2})\exp (i{\varphi }_{2}{M}_{1})\exp (i{\varphi }_{3}{M}_{2})=\exp [i({\gamma }_{1}{M}_{1}+{\gamma }_{2}{M}_{2}+{\gamma }_{3}{M}_{3})],\end{array}$$

where the two sets *ϕ*_*i*_ and *γ*_*i*_ (*i* = 1, 2, 3) are in one-to-one correspondence^26^. Eq. (8) can be used for generators of the *SU*(2) group. Now we focus on the decoherence-free subspace in the triple quantum dot, and solve Eq. (8) by setting *γ*_1_ = *αa*, *γ*_2_ = *αb*, *γ*_3_ = 0, where *a*^2^ + *b*^2^ = 1. It is easy to verify that the powers of *M*_*i*_ satisfy

9 $$\begin{array}{l}{M}_{i}^{2n}=I-{\rm{\Delta }}(i),\,{M}_{i}^{2n+1}={M}_{i},\\ {(a{M}_{1}+b{M}_{2})}^{2n}={(a{M}_{1}+b{M}_{2})}^{2},\\ {(a{M}_{1}+b{M}_{2})}^{2n+1}=a{M}_{1}+b{M}_{2},\end{array}$$

where *n* is a positive integer, *I* is the three dimensional identity matrix, and Δ(*i*) is a matrix whose *i*th diagonal matrix element is one and all other elements are zeros. Based on the above properties, we can derive an exact matrix equation

10 $$\begin{array}{c}(\begin{array}{ccc}\cos \,{\varphi }_{2} & i\,\sin \,{\varphi }_{2}\,\cos \,{\varphi }_{3} & -\sin \,{\varphi }_{2}\,\sin \,{\varphi }_{3}\\ i\,\cos \,{\varphi }_{1}\,\sin \,{\varphi }_{2} & \cos \,{\varphi }_{1}\,\cos \,{\varphi }_{2}\,\cos \,{\varphi }_{3}-\,\sin \,{\varphi }_{1}\,\sin \,{\varphi }_{3} & i\,\cos \,{\varphi }_{1}\,\cos \,{\varphi }_{2}\,\sin \,{\varphi }_{3}+i\,\sin \,{\varphi }_{1}\,\cos \,{\varphi }_{3}\\ -\sin \,{\varphi }_{1}\,\sin \,{\varphi }_{2} & i\,\sin \,{\varphi }_{1}\,\cos \,{\varphi }_{2}\,\cos \,{\varphi }_{3}+i\,\cos \,{\varphi }_{1}\,\sin \,{\varphi }_{3} & -\sin \,{\varphi }_{1}\,\cos \,{\varphi }_{2}\,\sin \,{\varphi }_{3}+\,\cos \,{\varphi }_{1}\,\cos \,{\varphi }_{3}\end{array})\\ =\,(\begin{array}{ccc}1+{a}^{2}(\cos \,\alpha -1) & ia\,\sin \,\alpha  & ab(\cos \,\alpha -1)\\ ia\,\sin \,\alpha  & \cos \,\alpha  & ib\,\sin \,\alpha \\ ab(\cos \,\alpha -1) & ib\,\sin \,\alpha  & 1+{b}^{2}(\cos \,\alpha -1)\end{array}),\end{array}$$

representing a system of nine nonlinear equations, among which only three equations are independent. The independent equations determine *ϕ*_1_ = *ϕ*_3_ and

11 $$\begin{array}{l}\tan \,{\varphi }_{1}=b\,\tan \,\frac{\alpha }{2},\,\sin \,\frac{{\varphi }_{2}}{2}=a\,\sin \,\frac{\alpha }{2}.\end{array}$$

Therefore angles *ϕ*_1_ and *ϕ*_2_ can be expressed in terms of *α*, *a* and *b*. Substituting Eq. (8) to *U*_x_(*g*, *δε*_d_, *θ*), we obtain

12 $$\begin{array}{l}{U}_{{\rm{x}}}(g,\delta {\varepsilon }_{{\rm{d}}},\theta )=\exp (i{\beta }_{1}\delta {\varepsilon }_{{\rm{d}}}{H}_{{\rm{leak}}}^{0})\exp (\,-\,i{\beta }_{2}{H}_{{\rm{x}}}^{0})\exp (i{\beta }_{1}\delta {\varepsilon }_{{\rm{d}}}{H}_{{\rm{leak}}}^{0}),\end{array}$$

where ${H}_{{\rm{leak}}}^{0}$ $({H}_{{\rm{x}}}^{0})$ is defined by ${H}_{{\rm{leak}}}=\delta {\varepsilon }_{{\rm{d}}}{H}_{{\rm{leak}}}^{0}$ (${H}_{{\rm{x}}}=g{H}_{{\rm{x}}}^{0}$). The parameter constraints are

13 $$\begin{array}{ccc}{\beta }_{1} & = & -\frac{1}{\delta {\varepsilon }_{{\rm{d}}}}\arctan (\frac{\delta {\varepsilon }_{{\rm{d}}}}{\sqrt{{g}^{2}+\delta {\varepsilon }_{{\rm{d}}}^{2}}}\,\tan \,\frac{\frac{\theta }{2g}\sqrt{{g}^{2}+\delta {\varepsilon }_{{\rm{d}}}^{2}}}{2}),\\ {\beta }_{2} & = & 2\arcsin (\frac{g}{\sqrt{{g}^{2}+\delta {\varepsilon }_{{\rm{d}}}^{2}}}\,\sin \,\frac{\frac{\theta }{2g}\sqrt{{g}^{2}+\delta {\varepsilon }_{{\rm{d}}}^{2}}}{2}),\end{array}$$

which are obtained from Eq. (11). By reversing Eq. (12) we obtain the ideal gate operator with respect to *x* axis,

14 $$\begin{array}{l}{U}_{{\rm{ix}}}({\beta }_{2})\,:=\exp (\,-\,i{H}_{{\rm{x}}}^{0}{\beta }_{2})=\exp (\,-\,i{\beta }_{1}\delta {\varepsilon }_{{\rm{d}}}{H}_{{\rm{leak}}}^{0}){U}_{{\rm{x}}}(g,\delta {\varepsilon }_{{\rm{d}}},\theta )\exp (\,-\,i{\beta }_{1}\delta {\varepsilon }_{{\rm{d}}}{H}_{{\rm{leak}}}^{0}),\end{array}$$

and eliminate the leakage *H*_leak_. Notice that constrains (13) hold for any magnitude of fluctuation *δε*_d_. Therefore, one can expand constrains (13) about any specific *δε*_d_. In fact, factor *δε*_d_ can be completely removed in the Taylor expansion of the above constrains. For example, when *δε*_d_ is relatively small compared to *g*, the Taylor expansion of constraints (13) in terms of (*δε*_d_/*g*) can be given by

15 $$\begin{array}{rcl}{\beta }_{1} & \approx  & -\frac{1}{g}\,\tan \,\frac{\theta }{4}+\frac{1}{24g}(8{\tan }^{3}\frac{\theta }{4}-3\theta {\tan }^{2}\frac{\theta }{4}+12\,\tan \,\frac{\theta }{4}-3\theta ){(\frac{\delta {\varepsilon }_{{\rm{d}}}}{g})}^{2}+O({(\frac{\delta {\varepsilon }_{{\rm{d}}}}{g})}^{4}),\\ {\beta }_{2} & \approx  & \frac{\theta }{2}+\frac{1}{4}(\theta -4\,\tan \,\frac{\theta }{4}){(\frac{\delta {\varepsilon }_{{\rm{d}}}}{g})}^{2}+O({(\frac{\delta {\varepsilon }_{{\rm{d}}}}{g})}^{4}).\end{array}$$

So, approximately one has

16 $${\beta }_{1}^{^{\prime} }=-\frac{1}{g}\,\tan \,\frac{\theta }{4},{\beta }_{2}^{^{\prime} }=\frac{\theta }{2}.$$

Actually, Eq. (16) correspond to the previous results^19^. If one applies Eq. (16) to perform an x-rotation through ${\beta }_{2}^{^{\prime} }$, only the values of *θ* and *g* are required to be set. Furthermore, the error scales of above approximate control conditions can be given by

17 $$\begin{array}{ll} & \exp (-i{\beta }_{1}^{^{\prime} }\delta {\varepsilon }_{{\rm{d}}}{H}_{{\rm{leak}}}^{0}){U}_{{\rm{x}}}(g,\delta {\varepsilon }_{{\rm{d}}},\theta )\exp (-i{\beta }_{1}^{^{\prime} }\delta {\varepsilon }_{{\rm{d}}}{H}_{{\rm{leak}}}^{0})\\ = & \exp \{-i[{\beta }_{1}\,g(\frac{\delta {\varepsilon }_{{\rm{d}}}}{g})+i{F}_{1}\,g{(\frac{\delta {\varepsilon }_{{\rm{d}}}}{g})}^{3}]{H}_{{\rm{leak}}}^{0}\}{U}_{{\rm{x}}}\exp \{-i[{\beta }_{1}\,g(\frac{\delta {\varepsilon }_{{\rm{d}}}}{g})+i{F}_{1}\,g{(\frac{\delta {\varepsilon }_{{\rm{d}}}}{g})}^{3}]{H}_{{\rm{leak}}}^{0}\}\\ = & \exp [i{F}_{1}\,g{(\frac{\delta {\varepsilon }_{{\rm{d}}}}{g})}^{3}{H}_{{\rm{leak}}}^{0}]\exp (\,-\,i{\beta }_{2}{H}_{{\rm{x}}}^{0})\exp [i{F}_{1}\,g{(\frac{\delta {\varepsilon }_{{\rm{d}}}}{g})}^{3}{H}_{{\rm{leak}}}^{0}]\\ \approx  & \exp [-i{\beta }_{2}^{^{\prime} }{H}_{{\rm{x}}}^{0}-i{F}_{2}{(\frac{\delta {\varepsilon }_{{\rm{d}}}}{g})}^{2}{H}_{{\rm{x}}}^{0}]+i{F}_{1}\,g{(\frac{\delta {\varepsilon }_{{\rm{d}}}}{g})}^{3}[{H}_{{\rm{leak}}}^{0}{U}_{{\rm{ix}}}+{U}_{{\rm{ix}}}{H}_{{\rm{leak}}}^{0}]+O({(\frac{\delta {\varepsilon }_{{\rm{d}}}}{g})}^{4}),\end{array}$$

where *F*_1_ and *F*_2_ denote the coefficients of (*δε*_d_/*g*)^2^ in the expansion of *β*_1_ and *β*_2_ respectively. The last step in the above expression is also based on Taylor expansion. Seen from Eq. (17), if the x-rotation is performed using Eq. (16), the terms in relation with computational and leakage error scale as (*δε*_d_/*g*)^2^ and (*δε*_d_/*g*)^3^ respectively. Therefore, the probability of computational error and leakage error scale as (*δε*_d_/*g*)^4^ and (*δε*_d_/*g*)^6^ respectively^19^. It is worth mentioning that the implementation of objective rotation angle *β*′_2_ depends on *θ* which is the multiplier of *g* and *t*_*x*_. Then one can enlarge *g* and shorten *t*_*x*_ to further suppress *δε*_d_/*g* for the same given *β*′_2_. Besides above example, one can obtain expansions about other points rather than *δε*_d_/*g* = 0 by constraints (13). Due to the mathematical form of the constrains, a singular point exists. Eq. (16) indicate that the value of ${\beta }_{2}^{^{\prime} }$ can be arbitrarily chosen except *π*. When ${\beta }_{2}^{^{\prime} }=\pi$, *θ*/4 = *π*/2 and tan(*θ*/4) goes to infinity. This can be solved by applying two sequences when *β*_2′_ = *π*/2, which does not change the error scales.

## Numerical Results

**Figure 2:**
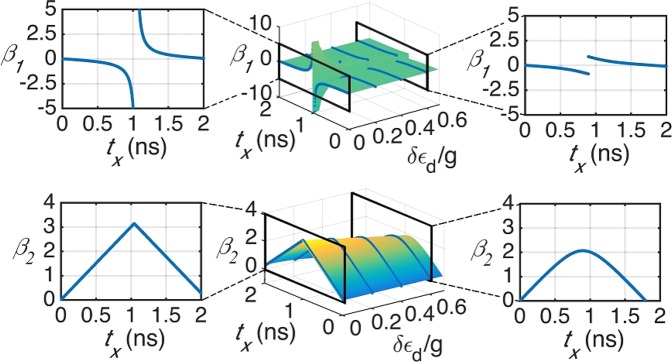
Surfaces determined by exact solutions in parameter space and four specific sectional graphs. The left (right) column shows the dependence of *β*_1_ or *β*_2_ on *t*_*x*_ when *δε*_d_/*g* is 0 (0.6). The middle column shows the surfaces. The upper panel is obtained by the first Eq. (13) and lower panel is obtained by the second Eq. (13). The curves when *δε*_d_/*g* = 0, 0.2, 0.4, 0.6 are also marked on the surfaces in order to visualize the basic trend.

We firstly show the parameter dependence of exact solution (13) by the surfaces in Fig. 2. We set *g*/*ℏ* = 3.0 GHz. *t*_*x*_ varies from 0 to 2.0 ns and *δε*_d_/*g* varies from 0 to 0.6. One of our main concern is the ability of rotating a qubit about x-axis under constrains (13), which is given by the domain of *β*_2_. Seen from the lower panel of Fig. 2, which is obtained by the second constrain (13), *β*_2_ exactly changes from 0 to 2*π* when *δε*_d_ = 0 (left subfigure in the lower panel). For the case when *δε*_d_ is bigger than 0, such as 0.6*g*/*ℏ* (right subfigure in the lower panel), one can only obtain a good approximation of the *δε*_d_ = 0 case when *t*_*x*_ is small. Besides, from the upper panel which is obtained by the first constrain (13), we can see that the control parameter *β*_1_ is also insensitive to noise, except the neighbour area of singular point *t*_*x*_ = 1.05 ns. The left (right) subfigure of the upper panel shows a sectional view of the case when *δε*_d_/*g* = 0 (0.6). In general, one can conclude from the above results that the circuit is insensitive to noise when control time is short. Also, an *x*-rotation of the CQ qubit through an arbitrary angle can be performed under constrains (13) with one or two sequences (14).

**Figure 3:**
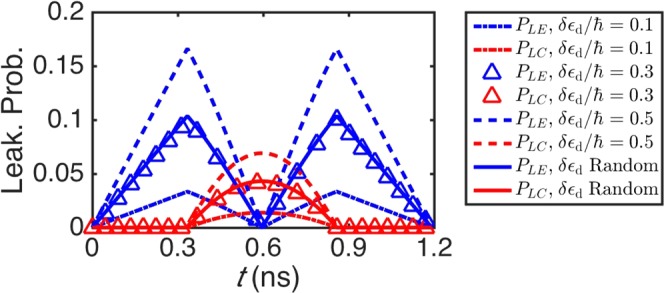
The probability of leakage error *P*_*LC*_ (red) and *P*_*LE*_ (blue) under quasistatic noise approximation. Each condition of the curve is demonstrated by the legend on the right. The dash-dotted, triangle-dotted, and dashed curves show the results when *δε*_d_/*ℏ* = 0.1, 0.3, 0.5 GHz respectively. Solid lines show the average leakage probabilities of the quasistatic noise channels with random magnitude varying from 0.1 to 0.5 GHz.

Next, we show a numerical analysis of the leakage errors of our whole *x* rotation scheme, with the probability of leakage errors defined by *P*_*LC*,*LE*_ = |〈*L*|*U*|*C*, *E*〉|^2^ ^19^. We consider the cases of different types of system noise, i.e., different *δε*_d_s, and the results are given by Fig. 3. *g*/*ℏ* is also set to be 3.0 GHz and *β*_2_ is set to be *π*/2. The control parameter *β*_1_ and *θ* of the circuit (14) applied in all the cases are obtained by solving constrains (13) under above conditions together with the assumption that *δε*_d_/*ℏ* is 0.3 GHz, no matter how it is set in the simulation of a specific evolution. The leakage probabilities are obtained from circuit (14) under quasistatic noise assumption, when system *δε*_d_/*ℏ* (the one used for the simulation of the evolution by the circuit) is 0.1, 0.3, 0.5, or randomly varies from 0.1 to 0.5 GHz in a multi-channel case. The results of *δε*_d_/*ℏ* = 0.1, 0.3, 0.5 GHz are represented by dash-dotted, triangle-dotted, and dashed curves respectively. The solid lines display the results of leakage probabilities of the multi-channel case. In such a case, more than one noise channel is calculated. The *δε*_d_/*ℏ* of a certain channel is static, but randomly varies from 0.1 to 0.5 GHz for different channels. The solid curves in Fig. 3 are obtained by averaging over 100 noise channels. The final leakage probabilities *P*_*LC*_ (red curves) and *P*_*LE*_ (blue curves) are both below 1.43 × 10^−4^. The infidelity 1 − *F* (*F* is the average gate fidelity^27,28^) in 2D logical subspace of above cases are below 9.76 × 10^−6^ which gives the order of the computational error. For this part of results, two conclusions can be drawn. One is that the effectiveness of the circuit (14) does not depend upon an exact knowledge of the noise amplitude. This is indicated by the good performance of the circuit (14) whose parameters are obtained by assuming a fixed value of *δε*_d_/*ℏ* (0.3 GHz), rather than directly using the noise information of corresponding numerical simulation of evolution (system *δε*_d_/*ℏ* = 0.1, 0.5 GHz and random). As for other assumptions of the value of *δε*_d_/*ℏ*, one can also adjust the control parameters of circuit (14) properly according to constrains (13) for a required elimination of leakage. The other is that the changing character of the leakage probabilities of the multi-channel case coincides with that of the single channel case whose system *δε*_d_/*ℏ* is set to be the average of the random one, indicating that multi-channel average will not disturb the effectiveness of the whole process. Therefore, one could expect a stability of circuit (14) in the potential applications.

A noise-free version of constrains (13) requires Taylor expansion. So we numerically analyze an example of such approximation. We consider circuit (14) under Eq. (16). *β*′_2_ is set to be *π*/2. In order to evaluate a case when *δε*_d_/*g* is not relatively big, we set *g*/*ℏ* = 0.5 GHz. By replacing the *β*_1_ and *β*_2_ in circuit (14) by *β*′_1_ and *β*′_2_, one can calculate *P*_*LC*,*LE*_. *θ* and *β*′_1_ can be obtained by solving Eq. (16) in the above setup. *δε*_d_ is also assumed to be constant in one rotation circuit. The results are taken by averaging over different channels where *δε*_d_/*ℏ* randomly varies from 0.15 to 0.45 GHz. The final values of *P*_*LC*,*LE*_ are 1.63 × 10^−4^ and 4.01 × 10^−4^, and the infidelity is 2.64 × 10^−5^. These numbers support the effectiveness of the approximation. Also, we consider the case when *δε*_d_/*ℏ* in circuit (14) changes with time frequently. The simulation is performed by decomposing each exponential operator into a sufficient large number of pieces and *δε*_d_/*ℏ* randomly varies from piece to piece in the range of 0.15 to 0.45 GHz. The final values of average *P*_*LC*,*LE*_ are 7.85 × 10^−4^ and 1.05 × 10^−3^ and the infidelity is 2.92 × 10^−4^, which also shows a insensitivity to the noise.

## Estimation of Noise Strength

Based on our exact formula, we can estimate the strength of *δε*_d_. For a CQ qubit under noise *δε*_d_, an ideal *x* rotation with angle *β*_2_ is generated by experimental parameters *β*_1_, *θ*, *g* and *δε*_d_ in terms of the constraints (13). In semiconducting quantum dots, gate operations are implemented by microwave pulses so that *θ* can be modulated by the pulse width, and *g* is determined by tunnel couplings *t*_A,B_. The spectrum of the noise *δε*_d_ in range of 5 kHz to 1 MHz has been shown by Hahn echo curves^21^. Here our derivation suggests a new perspective to look into the noise *δε*_d_. An estimation of *δε*_d_ can be done by following steps. (i) Prepare an initial state, for example |*C*〉. (ii) Perform the three operations on the right side of Eq. (14) with given *g*, *β*_1_ and *θ* which has no limitation other than the first Eq. (13). A good approximation can be obtained when *g* is set to be sufficiently large, such as the first Eq. (16). The resultant operation in the logical subspace is an ideal *x* rotation. (iii) Measure the output state, and then *β*_2_ can be given. (iv) Substitute *g*, *θ* and the measurement result of *β*_2_ to the second Eq. (13), then *δε*_d_ is estimated. Our analysis in the previous sections indicates that the first Eq. (13) is mainly in relation with leakage error. So the estimation based on the second Eq. (13) in the logical subspace is little affected by the imperfect control of *β*_1_. In experiments on semiconducting quantum dots, state initialization and readout take about 4 ms to 5 ms, and state manipulation needs about 1 ms^21^. Therefore our estimation is allowed to be performed and repeated for several times and an effective strength curve of *δε*_d_ in the time domain can be concluded.

## Arbitrary Single Qubit Rotation

An arbitrary leakage-free gate can be generated by three ideal *x* and *z* rotations. While the ideal *x* rotation is given by (14), in what follows, we will first show how to generate the ideal *z* rotation. Let us start with the experimentally available *U*_z_(*ε*_q_, *δε*_d_, *ϕ*) in Eq. (5). By using the commutation relation [*H*_z_, *H*_leak_] = 0, *U*_z_ can be simply decomposed into

18 $${U}_{{\rm{z}}}({\varepsilon }_{{\rm{q}}},\delta {\varepsilon }_{{\rm{d}}},\phi )=\exp [-i{H}_{{\rm{z}}}({\varepsilon }_{{\rm{q}}})\phi /{\varepsilon }_{{\rm{q}}}]\exp [-i{H}_{{\rm{leak}}}(\delta {\varepsilon }_{{\rm{d}}})\phi /{\varepsilon }_{{\rm{q}}}].$$

Consequently, the leakage-free *z* rotation can be realized by

19 $${U}_{{\rm{iz}}}(\phi )\,:\,=\exp (-\,i{H}_{{\rm{z}}}^{0}\phi )={U}_{{\rm{z}}}({\varepsilon }_{{\rm{q}}},\delta {\varepsilon }_{{\rm{d}}},\phi )\exp (-\,i{H}_{{\rm{leak}}}^{0}\delta {\varepsilon }_{{\rm{d}}}\phi ^{\prime} ).$$

${H}_{{\rm{z}}}^{0}$ is given by ${H}_{{\rm{z}}}={\varepsilon }_{{\rm{q}}}{H}_{{\rm{z}}}^{0}/2$ and *φ*′ = −*φ*/*ε*_q_. Also, $\exp (\,-\,i{H}_{{\rm{leak}}}^{0}\delta {\varepsilon }_{{\rm{d}}}\phi \text{'})$ can be implemented by setting *ε*_q_ = 0. Equation (19) shows that only two different gates with the same *δε*_d_ are needed for the implementation of an ideal *z* rotation. It does not require the detail of *δε*_d_ as well.

**Figure 4:**
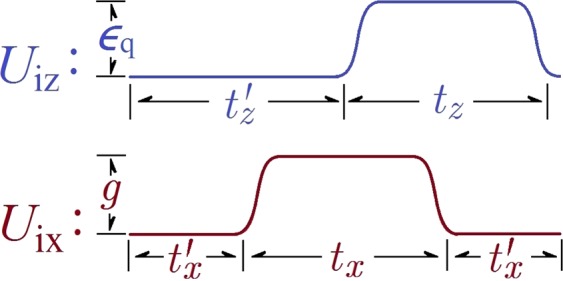
Circuits for generating *U*_ix_ and *U*_iz_. The magnitude of applied pulses are *ε*_q_ and *g*. The operation time *t*_*z*_ (*t*′_*z*_) is given through angle *φ* (*φ*′) and detuning parameter *ε*_q_, and *t*_*x*_ (*t*′_*x*_) is given by angles *θ* (*β*_1_) and the coupling parameter *g*.

In general, it is well-known that an arbitrary leakage-free rotation for a single qubit can be implemented by combining *U*_ix_ and *U*_iz_, *i*.*e*., three experimentally-available rotations for *x* axis and two for *z* axis, as sketched in Fig. 4. In the above rotation scheme, a conjugate of operator can also be implemented by circuits composed of the *su*(2) generators that correspond to the short-time evolution when *ε*_q_ or *g* is set large enough. For example, when *g* = *ε*_q_ = 12.0 GHz and noise is 0.3 GHz, *U*_z_(0, *δε*_d_,*φ*) can be inverted with infidelity 4.2 × 10^−3^.

## Conclusion

We provide an exact solution to elimination of leakage errors in a three-level quantum model using simple circuits of gates. The model comprises of two logical states and a leakage state, which can be used to describe a triple quantum dot system supporting a DFS. Encoding qubits in a DFS is a well-known strategy in error suppression for quantum computation, which attracts significant attentions because of its minimal overhead requirements. The concatenation of DFS and the circuits promises to give this approach a twofold resilience, against decoherence and stochastic leakage errors. In comparison with the previous work^19^, our formalism is based on finite rotations and *su*(2) subalgebra. Reasonable approximations for application can be obtained from the formula. For example, one will trace back to the results in ref.^19^ by assuming the noise is relatively small. We numerically analysis the parameter dependence of the exact solution, especially the affection of the noise. Numerical simulation shows that the performance of our circuits does not rely on an accurate knowledge of the noise, and is excellent in a multi-channel model when the noise strength randomly varies from channel to channel, indicating the stability of these circuits. Furthermore we propose an estimation of dipolar detuning control fluctuation to extract strength information of noise. The feasibility of our approach is ensured by the development of sophisticated experimental techniques^19,20^.

## Data Availability

The datasets generated and analysed during the current study are available from the corresponding author on reasonable request.

## References

[1] Loss D, DiVincenzo DP (1998). Quantum computation with quantum dots. Phys. Rev. A.

[2] Elzerman JM (2004). Single-shot read-out of an individual electron spin in a quantum dot. Nature.

[3] Petta JR (2005). Coherent manipulation of coupled electron spins in semiconductor quantum dots. Science.

[4] Hanson R, Kouwenhoven LP, Petta J, Tarucha RS, Vandersypen LMK (2007). Spins in few-electron quantum dots. Rev. Mod. Phys..

[5] Shulman MD (2014). Suppressing qubit dephasing using real-time Hamiltonian estimation. Nat. Commun..

[6] Veldhorst M (2015). A two-qubit logic gate in silicon. Nature.

[7] Wu L-A, Kurizki G, Brumer P (2009). Master equation and control of an open quantum system with leakage. Phys. Rev. Lett..

[8] Taylor JM (2005). Fault-tolerant architecture for quantum computation using electrically controlled semiconductor spins. Nat. Phys..

[9] Byrd MS, Lidar DA, Wu L-A, Zanardi P (2005). Universal leakage elimination. Phys. Rev. A.

[10] Motzoi F, Gambetta JM, Rebentrost P, Wilhelm FK (2009). Simple Pulses for Elimination of Leakage in Weakly Nonlinear Qubits. Phys. Rev. Lett..

[11] West JR, Fong BH (2012). Exchange-only dynamical decoupling in the three-qubit decoherence free subsystem. New. J. Phys..

[12] Ghosh J, Fowler AG, Martinis JM, Geller MR (2013). Understanding the effects of leakage in superconducting quantum-error-detection circuits. Phys. Rev. A.

[13] Hickman GT, Wang X, Kestner JP, Das Sarma S (2013). Dynamically corrected gates for an exchange-only qubit. Phys. Rev. B.

[14] Ghosh J (2013). High-fidelity controlled-*σ*^*z*^ gate for resonator-based superconducting quantum computers. Phys. Rev. A.

[15] Egger DJ, Wilhelm FK (2014). Optimized controlled-Z gates for two superconducting qubits coupled through a resonator. Supercond. Sci. Technol..

[16] Zahedinejad E, Ghosh J, Sanders BC (2015). High-Fidelity Single-Shot Toffoli Gate via Quantum Control. Phys. Rev. Lett..

[17] Wu L-A, Byrd MS, Lidar DA (2002). Efficient Universal Leakage Elimination for Physical and Encoded Qubits. Phys. Rev. Lett..

[18] Jing J (2015). Nonperturbative Leakage Elimination Operators and Control of a Three-Level System. Phys. Rev. Lett..

[19] Ghosh J, Coppersmith SN, Friesen M (2017). Pulse sequences for suppressing leakage in single-qubit gate operations. Phys. Rev. B.

[20] Friesen M, Ghosh J, Eriksson MA, Coppersmith SN (2017). A decoherence-free subspace in a charge quadrupole qubit. Nat. Commun..

[21] Kawakami E (2016). Gate fidelity and coherence of an electron spin in an Si/SiGe quantum dot with micromagnet. Proc. Natl. Acad. Sci..

[22] Eng K (2015). Isotopically enhanced triple-quantum-dot qubit. Sci. Adv..

[23] Das Sarma SR, Throckmorton E, Wu Y-L (2016). Dynamics of two coupled semiconductor spin qubits in a noisy environment. Phys. Rev. B.

[24] Schutjens R, Abu Dagga F, Egger DJ, Wilhelm FK (2013). Single-qubit gates in frequency-crowded transmon systems. Phys. Rev. A.

[25] Edmonds, A. R. *Angular Momentum in Quantum Mechanics*, (Princeton University Press, Fouth printing, 1996).

[26] Varshalovich, D. A., Moskalev, A. N. & Khersonskii, V. K. *Quantum Theory of Angular Momentum*, (World Scientific, Singapore, 1988).

[27] Pedersen LH, Møller NM, Mølmer K (2007). Fidelity of quantum operations. Phys. Lett. A.

[28] Ghosh J, Geller MR (2010). Controlled-NOT gate with weakly coupled qubits: Dependence of fidelity on the form of interaction. Phys. Rev. A.

